# Clinical Variability and Genotype–Phenotype Correlation in Spanish Patients with Type 1 Gaucher Disease: A Focus on Non-c.[1226A>G]; [1448T>C] Genotypes

**DOI:** 10.3390/ijms262010088

**Published:** 2025-10-16

**Authors:** Irene Serrano-Gonzalo, Francisco Bauza, Laura Lopez de Frutos, Isidro Arevalo-Vargas, Mercedes Roca-Espiau, Marcio Andrade-Campos, Esther Valero-Tena, Sonia Roca-Esteve, David Iniguez, Pilar Giraldo

**Affiliations:** 1 Fundación Española para el Estudio y Terapéutica de la Enfermedad de Gaucher y Otras Lisosomales (FEETEG), 50006 Zaragoza, Spain; ireneserranogonzalo@gmail.com (I.S.-G.); 811305@posta.unizar.es (I.A.-V.);; 2 Department of Biochemistry and Cellular and Molecular Biology, Universidad de Zaragoza, 50018 Zaragoza, Spain; 3 Instituto de Biocomputación y Física de Sistemas Complejos (BIFI), Universidad de Zaragoza, 50018 Zaragoza, Spain; francisco.bauza@bifi.es (F.B.); david.iniguez@bifi.es (D.I.); 4 Grupo Español de Enfermedades de Depósito Lisosomal de la SEHH (GEEDL), 50006 Zaragoza, Spain; 5 Instituto de Investigación Hospital del Mar, IMIM-Parc Salut Mar, 08003 Barcelona, Spain; 6 Fundación ARAID, 50018 Zaragoza, Spain

**Keywords:** Gaucher disease, genotype, phenotype, machine learning, bone disease, Parkinson disease

## Abstract

The clinical heterogeneity of type 1 Gaucher disease (GD1) underscores the limited correlation between the *GBA1* genotype and phenotype. This study examined GD1 patients from the Spanish Gaucher Disease Registry carrying heterozygous *GBA1* genotypes distinct from NM_000157: c.[1226A>G](N370S); [1448T>C](L444P). Among 374 patients with GD1, 195 (52.1%) had alternative heterozygous combinations, including variants corresponding to severe (37.9%) or moderate (42.1%) mutation, whereas only 20% patients harbored mild variants—all of them in combination with N370S. Descriptive statistics and predictive models based on logistic regression and decision trees were applied. Patients carrying N370S with a different L444P variant showed significantly higher rates of advanced bone disease (59.9%) compared to those with homozygous N370S (38.3%) or N370S; L444P (41.0%) (*p* = 0.002). Decision tree analysis identified the bone marrow burden score (S-MRI) as the strongest predictor of osteopenia/osteoporosis at diagnosis. Genotype also emerged as a key discriminator for Parkinson’s disease: patients with non-N370S; L444P genotypes showed a markedly higher likelihood of developing Parkinsonism. Overall, GD1 patients with genotypes other than N370S; L444P present more severe phenotypes, particularly with greater skeletal involvement and neurological complications. These findings highlight the importance of genotype stratification and predictive modeling in improving risk assessment and clinical management in GD1.

## 1. Introduction

Type 1 Gaucher disease (GD1, OMIM #230800) is a rare lysosomal storage disorder characterized by a wide spectrum of clinical manifestations, ranging from asymptomatic presentations to severe multi-organ involvement. It is caused by biallelic pathogenic variants in the *GBA1* gene (MIM*606463), which encodes the lysosomal enzyme β-glucocerebrosidase [1]. Deficiency of this enzyme leads to the accumulation of glucocerebroside in macrophages, resulting in diverse systemic complications [2].

Despite substantial research, the correlation between *GBA1* genotypes and clinical phenotypes remains incomplete, limiting our ability to predict disease severity and progression based solely on genetic information [3]. Numerous studies have reported intrafamilial variability among individuals with the same genotype, including siblings, underscoring the unpredictable nature of clinical outcomes [4]. Similarly, large registries and case series have documented significant phenotypic diversity among patients with identical *GBA1* variants [5].

In general, the c.1226A>G variant is associated with milder disease, particularly in homozygous individuals, whereas homozygosity for the c.1448T>C variant is linked to neuronopathic forms of Gaucher disease [6]. In Spain, the most common *GBA1* genotype is the heterozygous association c.1226A>G; however, other, less common genotypes are also present and exhibit considerable phenotypic variability [7]. The relationship between the severity of enzyme dysfunction and clinical expression continues to be a subject of interest in predicting individual disease trajectories. However, the extensive allelic heterogeneity and the presence of rare variants hinder the establishment of robust genotype–phenotype correlations [8].

This complexity suggests that additional genetic, epigenetic, and environmental modifiers likely contribute to the observed clinical heterogeneity in GD1. Several studies have explored the influence of other genetic loci [9], epigenetic mechanisms, and biomarkers identified through proteomic, lipidomic, and metabolomic analyses to better understand phenotypic expression [10,11,12]. Furthermore, the genotyping of *GBA1* is complicated by the presence of a highly homologous pseudogene, which increases the risk of partial recombination and can result in misclassification of alleles, particularly when using next-generation sequencing (NGS) platforms [13].

Recent advances in machine learning and complex network analysis offer promising tools for deciphering the intricate relationships between genetic, clinical, and biochemical variables in GD1. These approaches enable the analysis of large, multidimensional datasets and may improve diagnostic precision, guide treatment strategies, and enhance prognostic predictions. Their application in rare diseases such as GD1 is especially valuable as they can provide novel insights into the mechanisms driving disease variability [14,15,16,17].

In this context, the present study aims to assess the clinical characteristics at diagnosis and during follow-up in a cohort of Spanish GD1 patients with heterozygous *GBA1* genotypes other than the common NM_000157: c.[1226A>G]; [1448T>C]. By focusing on this subgroup, we aim to improve our understanding of the genotype–phenotype correlations and contribute to the broader knowledge of factors influencing clinical variability in GD1.

## 2. Results

### 2.1. Patients Genotype and Characteristics

A total of 358 patients with GD1 were included in this study. Of these, 308 had a heterozygous genotype in the *GBA1* gene. Specifically, 113 patients carried the c.[1226A>G]; [1448T>C] genotype, while 195 had a heterozygous c.[1226A>G] genotype with a second variant distinct from c.[1448T>C]. Demographic data and clinical characteristics are detailed in Table 1.

When we analyze the group of patients with heterozygous c.[1226A>G] genotype with a second variant distinct from c.[1448T>C], the gender ratio is 1:0.8 (♂:♀), and almost half of the patients (55.41%) were diagnosed after the year 2000. The mean age at diagnosis was 24.7 years old (range: 1.0–77.0), and the mean age at start of therapy was 27.9 years old (range: 1.0–74.0). Thirty-eight patients (19.5%) were previously fully splenectomized.

During follow-up, 8 patients (4.8%) developed monoclonal gammopathy of undetermined significance (MGUS), 10 patients (5.1%) developed other malignancies, and 12 patients (6.1%) developed Parkinson’s disease. Of the 180 patients questioned, 20 patients (10.2%) were diagnosed with cholelithiasis, while 31 patients (17.2%) presented with other comorbidities. Fourteen patients (8.0%) had died by the time of the study. Regarding bone disease, 44 patients (22.51%) had advanced bone disease with skeletal complications, and 62 patients (50.8%) exhibited varying degrees of bone mineral density (BMD) loss.

In the Spanish Registry of GD, the most frequent allelic variant is c.[1226A>G] (75% of alleles), followed by c.[1448T>C] (18.5%). In Spanish GD patients, the most frequent genotype is heterozygous c.[1226A>G] (N370S) combined with c.[1448T>C] (L444P). However, 195 patients (52.13%) had a *GBA1* heterozygous genotype other than NM_000157: c.[1226A>G] (N370S); [1448T>C] (L444P). These alternative genotypes are detailed in Table 2, with the new and classic protein nomenclature, and stratified according to functional severity.

### 2.2. Bone Involvement

A strong correlation between prior splenectomy and the presence of bone disease was observed (*p* = 0.005). In terms of genotype–phenotype correlation, patients with *GBA1* genotypes other than c.[1226A>G]; [1448T>C] showed a significantly higher frequency of severe bone disease at diagnosis (*p* = 0.05) in terms as greater bone marrow infiltration and skeletal complications compared to patients homozygous for c.[1226A>G] and heterozygous for c.[1226A>G]; [1448T>C] (*p* = 0.017).

**Figure 1:**
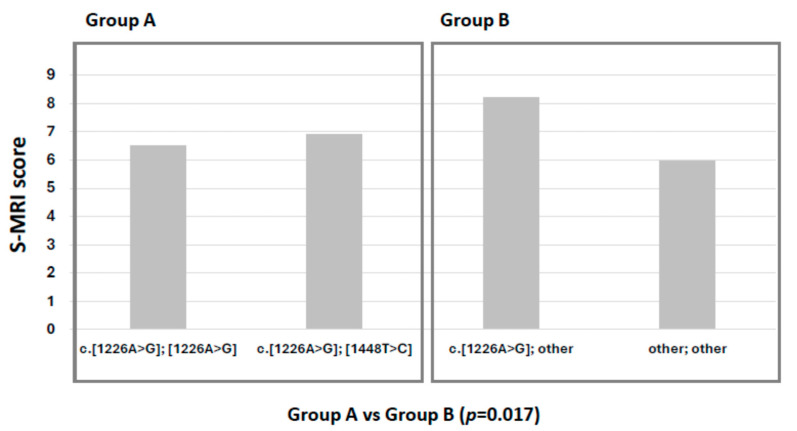
Degree of bone marrow infiltration and complications according to S-MRI score in relation to genotype.

In our cohort, patients heterozygous for c.1226A>G with another variant (excluding c.1448T>C), as well as those with other genotypes (group B), exhibited significantly greater bone marrow infiltration and complications compared to patients with homozygous c.1226A>G (N370S/N370S) and heterozygous c.[1226A>G]; [1448T>C] (N370S/L444P) genotypes (group A) (*p* = 0.017) (Figure 1).

The N370S; N370S cohort included 53 patients, only 4 of whom were splenectomized. A few patients developed bone complications, and none developed PD.

The other group included 17 patients with generally mild variants with non-neurological symptoms, including mutations such as R463H, R496H, N188K, S13L, and R170C in a homozygous or heterozygous manner.

In addition, the severity of bone disease regarding gender was worse in males heterozygous for c.[1226A>G] with another variant (mean S-MRI: 10) vs. females (mean S-MRI: 6) (*p* < 0.001). In addition, there is a very clear relationship between bone mineral loss and S-MRI (*p*-value = 0.0001).

**Figure 2:**
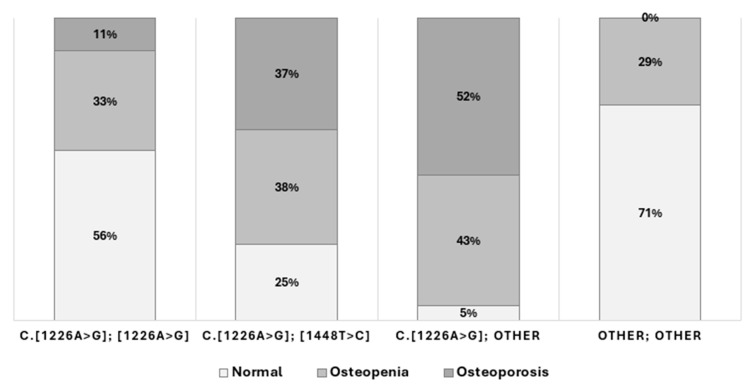
Distribution of bone mineral density in relation to genotype.

According to genotype, the presence of bone complications, such as infarct, necrosis, or fractures, was 59.9% for c.[1226A>G]; other different c.[1448T>C] vs. 38.3% for homozygous c.[1226A>G] and 41.0% for c.[1226A>G]; [1448T>C] (*p* = 0.002). These patients also had a significantly higher incidence of new bone crises during therapy (*p* = 0.0001). These groups demonstrated the highest degree of skeletal involvement. Moreover, patients homozygous for c.[1226A>G] (N370S) had a significantly lower prevalence of BMD loss compared to all other genetic subgroups (*p* = 0.003) (Figure 2). Moreover, a significant relationship was observed between bone mineral density loss and splenectomy patients (*p* = 0.0045), with a higher incidence in females (*p* = 0.023).

**Figure 3:**
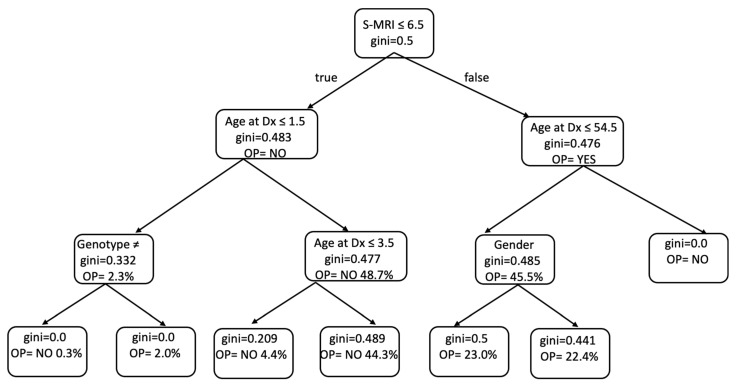
Simplified decision tree derived from the Random Forest model predicting bone mineral density loss in Gaucher disease. The most informative predictors were S-MRI score, age at diagnosis, and gender. Nodes indicate class (Normal vs. Osteo), and arrows indicate the dominant splits. Gini impurity was used for node splitting. This schematic displays the top decision paths for visualization; the full model performance is reported in the Supplementary Materials. ≠ genotype other than c.[1226A>G]; [1448T>C] vs. c.[1226A>G]; [1448T>C]. OP: osteopenia or osteoporosis; Gini index: statistical measure used to assess inequality in the distribution.

The predictive capacity of machine learning models to estimate the risk of severe bone involvement in Gaucher disease was further explored using a decision tree analysis (Figure 3). The model identified the S-MRI score as the most influential variable in determining bone status at diagnosis, with a threshold of 6.5 effectively separating patients with a higher probability of developing osteopenia or osteoporosis. Among those with lower S-MRI scores (≤6.5), age at diagnosis and genotype (excluding the common c.1226A>G or c.[1226A>G]; [1448T>C] variants) were key discriminators. In contrast, in patients with higher S-MRI scores, gender and age also contributed significantly to classification. The model’s discriminatory power supported the weight of the S-MRI score as the primary classifier (Table 3).

### 2.3. Development of Parkinson’s Disease

In the case of Parkinson’s disease, a significant relationship between the development of PD and genotypes other than homozygous c.[1226A>G] or heterozygous c.[1226A>G]; [1448T>C] is observed (*p* = 0.025). The very significant incidence of PD in patients with a family history of PD is noteworthy, independent of genotype (*p* = 0.00014). A relationship between PD and elevated ferritin levels can also be discerned (*p* = 0.02).

**Figure 4:**
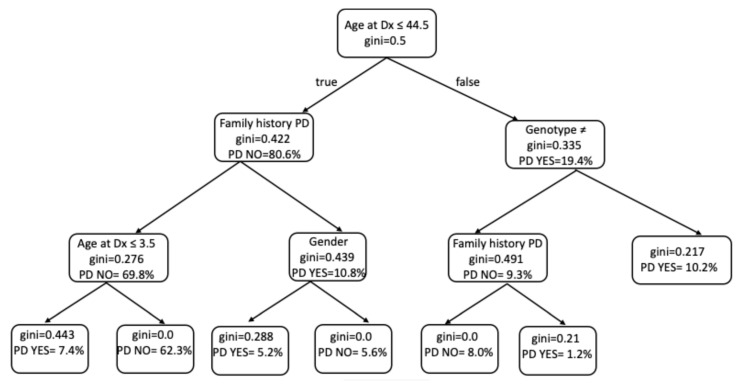
Simplified decision tree derived from the Random Forest model predicting Parkinson’s disease. Age at diagnosis, family history of PD, and genotype severity were the main contributors. Node indicates class (No-PD vs. PD). Gini impurity was used for node splitting. The diagram summarizes the dominant decision paths; complete model metrics are provided in Supplementary Material. ≠ genotype other than c.[1226A>G]; [1448T>C] vs. c.[1226A>G]; [1448T>C]. PD: Parkinson’s disease; Gini index: statistical measure used to assess inequality in the distribution.

Despite the limited number of patients who developed Parkinson’s disease (n = 14), the decision tree analysis (Figure 4) yielded meaningful insights into the variables most predictive of this outcome. Age at diagnosis was the primary node, with younger age (≤44.5 years) being associated with lower risk. Among these patients, the absence of a family history of Parkinson’s disease and younger diagnostic age (≤3.5 years) further stratified those at reduced risk. Conversely, in patients diagnosed after 44.5 years of age, genotype emerged as a key discriminator: individuals with genotypes other than homozygous c.1226A>G or compound heterozygous c.[1226A>G]; [1448T>C] exhibited a substantially higher probability of developing Parkinson’s disease. Values across the tree support the relevance of genotype and family history as dominant variables (Table 4).

## 3. Discussion

Several international registries have been established to collect data from patients with Gaucher disease (GD), enabling the analysis of clinical, genetic, and therapeutic aspects across diverse populations. The first global registry (NCT00358943), launched in 1991 by the International Collaborative Gaucher Group (ICGG) with support from Genzyme-Sanofi, has provided valuable longitudinal data regardless of disease severity, treatment status, or therapeutic approach [18]. Similarly, the Gaucher Outcome Survey (GOS), funded by Shire-Takeda since 2010, has enrolled over 2000 patients as of early 2023, primarily from Israel and the United States, to assess the long-term safety and effectiveness of velaglucerase alfa and other GD therapies [19].

However, certain biases in the composition of these registries must be acknowledged. In GOS, nearly half of the patients were from Israel, where a higher prevalence of genotypes associated with mild disease phenotypes is well documented. A study by Zimran et al., which followed 103 untreated GD1 patients for over six decades, revealed a notably benign course, with mild hepatosplenomegaly, minimal rates of splenectomy, and no clinical bone involvement [20]. These findings underscore the significant influence of genotype on phenotypic expression and disease course.

In this context, Gaucher disease has emerged as a model within the lysosomal disorder group, owing to the availability of robust clinical and genetic datasets, validated biomarkers, and therapeutic diversity. Nowadays, the application of artificial intelligence (AI) and machine learning techniques to these datasets has proven especially valuable in identifying patterns and predicting clinical outcomes. Recent studies have successfully employed such methodologies in the diagnosis of rare diseases, in patient stratification using next-generation sequencing (NGS) [21], and in multi-omics integration [22].

Specifically in GD, machine learning models have shown high performance in identifying undiagnosed patients using real-world clinical and administrative data, significantly reducing diagnostic delays [23,24,25]. Our group has contributed to this field by integrating real-time registry data and biobank resources, allowing for in-depth analyses of clinical–biochemical correlations and the development of predictive models for disease-specific outcomes, such as skeletal complications [15,17], as well as for the identification of novel biomarkers [14].

Despite the extensive data generated, genotype–phenotype correlation in GD remains incomplete. This is partly due to the wide range of *GBA1* variants, the differential genotype distribution across populations, and the possible epistatic effects of other genetic or epigenetic modifiers that remain poorly understood [26]. The Spanish population, with its rich genetic diversity and complex historical admixture, offers an opportunity to explore these associations [27]. In our cohort, the analysis of genotypes—particularly heterozygous combinations other than c.[1226A>G]; c.[1448T>C]—revealed a more severe clinical profile. This observation, not clearly established in previous international studies, highlights the importance of population-specific analyses.

Related to the discussion about other modifiers of the phenotype in GD, both non-modifiable alterations, such as other associated genetic alterations, and epigenetic modifiers are the subject of broad debate [26,28,29]. Some of the other concomitant external factors, such as infections, lifestyle habits, and environmental toxins, are modifiable, but these factors have not been included in this study. They could, however, be considered in future studies involving machine learning methods.

The application of AI-driven predictive models in our analysis further revealed genotype-associated clinical features, such as an increased risk of Parkinson’s disease and more severe skeletal involvement, including a higher prevalence of bone mineral loss, in certain genotypic subgroups [30,31]. Notably, male patients with these genotypes exhibited significantly greater skeletal burden compared to their female counterparts—a sex-specific trend that we previously reported, although its underlying pathophysiological basis remains unclear [17]. Sex-based differences in bone maturation have been determined via MRI images of healthy subjects, primarily in the lumbar spine [30]. Other relevant factors include the protective estrogenic effects on bone metabolism in females, together with gender-related differences in growth and bone remodeling [31]. Thus, the male sex may act as a modifier of skeletal severity in Gaucher disease. However, note that while this is evident in our cohort, where it is described for the first time, our conclusion remains speculative. These findings align with previous reports, such as that by Basiri et al. (2023) [18], which showed that patients with compound heterozygous GBA1 genotypes (e.g., c.[1226A>G]; other) had a higher risk of osteonecrosis compared to those with the homozygous c.[1226A>G] genotype [18].

Furthermore, the prevalence of osteopenia and osteoporosis was significantly higher among patients with c.[1226A>G]; other genotypes (*p* < 0.001), reinforcing the hypothesis of a more severe skeletal phenotype in this subgroup. These results are consistent with earlier observations suggesting that certain—albeit less common—genotypic combinations may confer a higher risk of bone disease, including osteonecrosis and chronic skeletal complications [29,31].

In contrast, no significant differences were observed in the incidence of malignancies across genotypic subgroups, suggesting that cancer risk in GD may be independent of *GBA1* genotype [32].

However, our Random Forest analysis identified the c.[1226A>G]; other genotype as a strong predictor for the development of Parkinson’s disease, supporting growing evidence of genotype-associated neurological involvement. This is consistent with previous studies indicating an elevated risk of synucleinopathies in GD patients with compound heterozygous mutations, particularly those involving non-c.[1226A>G] alleles [33,34,35].

These insights underscore the value of combining detailed clinical registries with advanced computational approaches to unravel the multifactorial nature of Gaucher disease. Our data confirm that genotype plays a pivotal role in shaping clinical outcomes, particularly in the skeletal and neurological domains, and that male sex may act as an additional modifier of disease severity in specific genotypic contexts. Nevertheless, the scope and limitations of this study should be noted. All machine learning results only reflect internal validation on a single-center cohort (n = 195). We explicitly acknowledge that external generalization is limited and that estimates may still be optimistic despite cross-validation. Consequently, machine learning findings are interpreted as associative and hypothesis-generating; logistic regression remains the primary inferential analysis. Code and parameter settings used for training/validation are available from the corresponding authors upon reasonable request to facilitate reproducibility.

Finally, our findings highlight the importance of ongoing real-time data collection and multidisciplinary collaboration, including geneticists, clinicians, and computational biologists, in improving patient stratification and guiding personalized management strategies in rare diseases. Expanding this approach to larger and more diverse cohorts will be essential to validate these observations and further elucidate the underlying biological mechanisms.

## 4. Patients and Methods

### 4.1. Study Design and Population

The Spanish Gaucher Disease Registry (SGDR), established in 1996, collects demographic, clinical, genetic, laboratory, and imaging data on individuals diagnosed with Gaucher disease. To date, the registry includes data on 436 patients, of whom 374 have Type 1 Gaucher disease (GD1). For this study, we analyzed a subset of 358 GD1 patients with complete datasets.

All patients provided informed consent prior to inclusion in the registry. This study was conducted in accordance with Spanish biomedical research regulations (Law 14/2007) and the Declaration of Helsinki, and studies involving EU-based subjects must comply with the EU General Data Protection Regulation in the performance of all processing activities in connection with this study. All the patients included in the Spanish Registry have given their informed consent for the use of their data for research purposes. The scientific and ethics committees of Aragon (CEICA. PI11/00029 Date: 7 April 2011) and the FEETEG foundation approved this study. The blood samples were provided by the Biobank of the Aragon Health System, integrated in the Spanish National Biobanks Network (PT20/00112).

The anonymized dataset includes demographic data, clinical features at diagnosis, genetic findings, laboratory results, biomarker levels, and imaging assessments. Follow-up data include treatment regimens, occurrence of bone crises, development of malignancies and Parkinson’s disease, and survival outcomes.

### 4.2. Genetic Analysis

All patients underwent full *GBA1* gene sequencing using Sanger sequencing to identify biallelic variants. In cases where one pathogenic variant was unidentified, long-range or nested PCR using gene-specific primers was performed to amplify the functional gene segments. These products were then sequenced to detect novel mutations. For patients with compound heterozygosity, the phase was determined by parental genotyping.

To rule out the 55-bp deletion in exon 9, all patients homozygous for the c.1226A>G variant were reanalyzed, as previously described [36]. Additionally, in patients apparently homozygous for c.1448T>C, two separate fragments (250 and 223 bp), encompassing exons 9 and 10, respectively, were sequenced to exclude pseudogene interference and confirm the presence of the variant within the functional gene.

### 4.3. Biomarker Analysis

Biomarker analyses were performed at the FEETEG reference laboratory. Chitotriosidase activity was measured using the method described by Hollak et al. (1994), with the artificial substrate 4-methylumbelliferyl-β-D-N,N′,N″-triacetylchitotrioside [37]. CCL18/PARC, an inflammatory chemokine produced by monocytes and T lymphocytes, was quantified using immunoassay techniques, as described by Boot et al. (2004) [38]. Glucosylsphingosine (GluSph), the deacylated form of glucosylceramide, which was first described in 1982 by Nilsson and Svennerholm [39], appearing to specifically reflect the accumulation of substrates in GD, was measured using a validated liquid chromatography–tandem mass spectrometry (LC-MS/MS) method [40].

### 4.4. Bone Involvement Assessment

Bone involvement was evaluated using S-MRI scoring [41] and a structured bone marrow report as an assessment tool for assessing bone marrow infiltration severity by MRI [42]. Evaluations were performed by a radiologist specialized in bone marrow skeletal imaging and stratified into three severity levels: mild, moderate, and severe. The bone mineral density was evaluated by DXA, and patients were classified according to the World Health Organization’s (WHO) criteria [43]. In addition, the overall clinical disease severity at diagnosis assessment was measured using the GD1 Severity Scoring System (GD1-DS3), including three major domains (i.e., bone, hematology, and visceral) [44].

### 4.5. Statistical Analyses

Descriptive statistics were used to summarize the data. Quantitative variables are reported as means with interquartile ranges, while categorical variables are expressed as percentages. Pearson’s correlation coefficient was used to assess linear correlations, and Chi-square tests were employed for categorical variables.

Data preprocessing, analysis, and modeling were conducted using R software (version 3.6.2), incorporating various packages such as car, ggplot2, vcd, GGally, plyr, igraph, rpart, dplyr [45,46,47]. For numerical explanatory variables, the Brunner–Munzel test was used, assessing associations by dividing the numerical data into two groups based on the binary variable categories. Fisher’s exact test was applied for binary variables, and the generalized Fisher’s exact test was applied for multi-categorical variables.

### 4.6. Predictive Modeling

Following the exploratory analysis, we used statistical and machine learning models to characterize associations between clinical variables and the target outcomes (bone mineral density loss and Parkinson’s disease). The primary inferential model was logistic regression (reporting odds ratios and 95% CIs). Decision tree-based models (single trees and Random Forest) were used secondarily to probe potential non-linearities and interactions that are not easily captured by linear terms [48,49]. Continuous predictors were kept in their original scale; no Winsorization was applied. Categorical variables were dummy-coded. We restricted model complexity to match sample size and outcome prevalence (events-per-variable principle), using penalized logistic regression (L2) when appropriate. For tree-based models, we constrained maximum depth, minimum samples per split/leaf, and the number of estimators to limit variance. Class imbalance (notably for PD) was handled with class-weighting in the loss function; any resampling was only performed within the training folds to avoid leakage. To enhance interpretability, we provide permutation-based variable importance for Random Forest, partial dependence profiles for the top predictors (S-MRI, age at diagnosis, genotype severity, sex), and simplified decision tree schematics that summarize the dominant splits in the Random-Forest ensemble. These visuals are intended to explain patterns rather than to represent the full model. More details are included in the Supplementary Materials.

## 5. Conclusions

Our study reinforces the pivotal role of the *GBA1* genotype in shaping the clinical spectrum of Gaucher disease, particularly in relation to skeletal and neurological manifestations. Through the integration of real-world registry data, biobank resources, and machine learning models, we were able to identify specific genotypic combinations, especially those involving the c.[1226A>G]; other variants no c.[1448T>C], as strong predictors of disease severity and Parkinson’s disease risk.

These findings suggest the necessity of population-specific analyses, as genotypic distributions and their phenotypic consequences may vary significantly across cohorts. The Spanish population, characterized by high genetic diversity, provides a valuable context for uncovering less frequent but clinically relevant genotype–phenotype associations. Moreover, the observed sex-specific differences in skeletal involvement call for further investigation into potential biological modifiers of disease severity.

Overall, our results highlight the importance of combining clinical expertise with computational tools in the study of rare diseases. Ongoing data collection and interdisciplinary collaboration will be essential to refine patient stratification and advance personalized therapeutic strategies in Gaucher disease and other lysosomal disorders.

## 6. Highlights

Genotype–phenotype correlation remains incomplete in Gaucher disease, particularly for rare or compound heterozygous *GBA1* variants.

Machine learning models identified c.[1226A>G]; other variants as strong predictors of Parkinson’s disease and severe skeletal involvement.

Sex-specific trends were observed, with male patients showing greater skeletal burden in certain genotypic subgroups.

Population-specific analyses revealed associations not previously reported in larger international registries.

Integrating clinical registries, biobanks, and AI tools enhances our ability to predict disease outcomes and tailor patient management.

## Figures and Tables

**Table 1:** Demographic data and clinical characteristics.

		Genotype c.[1226A>G]; [1448T>C] (N = 113)	Genotype c.[1226A>G]; [Other] (N = 195)
DEMOGRAPHY			
Gender	♀/♂	55/58	92/103
Age at diagnosis (years)	♀ Mean (min–max) ♂ Mean (min–max)	28.2 (3–70) 27.4 (3–78)	26.8 (1–77) 22.7 (1–76)
Family history of Parkinson’s disease	Yes/No	16/97	27/168
Dead	Yes/No	15/107	14/180
Survival (years)	♀ Median (Q1–Q3) Mean (min–max) ♂ Median (Q1–Q3) Mean (min–max)	64.5 (50–68) 68 (54–82) 64.0 (35.5–76.5) 63 (32–78)	66.5 (58–70.5) 63.7 (56–73 47.0 (44–48) 55.9 (41.5–76.2)
CLINICAL CHARACTERISTICS			
Classification according to GD-DS3 score	Mild (%) Moderate (%) Severe (%)	♀; ♂ 41.8; 36.2 30.9; 39.6 27.3; 24.1	♀; ♂ 34.4; 40.0 30.0; 24.7 35.5; 34.3
Spleen removal	N (%)	20 (17.7)	38 (19.5)
Liver Volume	MN (multiples of normal)	1–2	1–3
Spleen Volume	MN multiples of normal)	5–13	5–15
Previous Bone Crisis	N (%)	26 (23.0)	59 (30.2)
IMAGE STUDIES			
S-MRI score	♀ Mean (min–max) ♂ Mean (min–max)	7.0 (0–17) 7.0 (0–24)	8.0 (0–24) 8.0 (0–25)
Bone Mineral Density (DXA) *	T score > −1 N (%) T score (−1 to −2.5) N (%) T score < −2.5 N (%)	64 (59.8) 28 (26.2) 15 (14.0)	60 (49.2) 33 (27.0) 29 (23.8)
ANALYTICAL DATA Mean (min–max)			
Hemoglobin (g/dL)		12.2 (4.5–17.0)	12.3 (6.5–18.5)
Leucocytes × 10^9^ (/L)		6.5 (1.3–23.0	6.2 (1.4–18.1)
Platelets × 10^9^ (/L)		106.1 (4.0–363.0)	93.1 (7.0–410)
Ferritin (mcg/L)		620.6 (9.5–1850.0	534.9 (14.0–2811.0)
Iron (mg/dL)		126.3 (13.0–230.2)	88.2 (24.0–1553.0)
Cholesterol (mg/dL)		145.7 (33.0–285.0)	147.6 (64–348)
Triglycerides (mg/dL)		141.0 (32.0–362.0)	146.8 (14.0–583.0)
Cholesterol HDL (mg/dL)		33.7 (14.0–234.0)	38.6 (11.0–297.0)
Cholesterol LDL (mg/dL)		86.0 (5.0–148.0)	87.4 (12.0–300.0)
AST (UI)		35.9 (9.2–89.0)	34.1 (12.0–100.0)
ALT (UI)		28.6 (4.0–82.0)	29.5 (6.0–142.0)
GGT (UI)		41.2 (2.4–297.0)	35.7 (7.0–174.0)
Bilirubin (mg/dL)		0.98 (0.11–4.0)	1.06 (0.17–4.70)
IgG (mg/dL)		1332.8 (523.0–2453.0))	1302.0 (565.0–2520.0)
IgA (mg/dL)		254.0 (22.0–765.0)	273.0 (90.0–2108.0)
IgM (mg/dL)		245.8 (49.0–949.0)	209.0 (25.0–532.0)
Monoclonal Gammopathy	Yes/No	3/103	8/167
Polyclonal Gammopathy	Yes/No	10/93	12/55
DIAGNOSIS			
GCase activity (nmol/mL/h)	Mean (min–max)	0.9 (0.1–2.0)	0.76 (0.1–2.2)
BIOMARKERS			
Chitotriosidase ChT (nmol/mL/h)	Mean (min–max)	11,322.0 (190.0–65,497.0)	10,290.0 (526.0–57,466.0)
CCL18/PARC (ng/mL)	Mean (min–max)	568.3 (52.0–3763.0)	716.6 (51.0–3895.0)
Glucosylsphingosine (ng/mL)	Mean (min–max)	100.5 (5.1–320.0)	126.7 (0.88–836.0)
FOLLOW-UP (5–26 YEARS)			
Age at start therapy (years)	Mean (min–max)	29.0 (2–69)	27.9 (1–74)
Cumulated time on therapy (years)	Mean (min–max)	19.3 (2–30)	20.7 (1–31)
New bone crisis	N (%)	8 (7.1)	17 (9.5)
Joint replacement	N (%)	6 (5.3)	14 (7.2)
Neoplasia	N (%)	9 (7.9)	10 (5.1)
Parkinson’s disease	N (%)	2 (1.7)	12 (6.1)
Other comorbidities **	N (%)	34 (30.0)	31(31.0)
Type of therapy	ERT N (%) SRT N (%) None N (%)	64 (56.6) 34 (30) 15 (13.4)	123 (63.1) 63 (32.3) 9 (4.6)

S-MRI: Spanish MRI bone marrow score; AST: aspartate aminotransferase liver enzyme; ALT: alanine aminotransferase liver enzyme; GGT: gamma-glutamyl transferase liver enzyme; CCL18/PARC: CC chemokine pulmonary and activation-regulated chemokine. * There were 107 patients with data in group c.[1226A>G]; [1448T>C], and 122 in group c.[1226A>G]; [other]. ** Cholelithiasis, AHT, Diabetes, Thyroid dysfunction.

**Table 2:** More frequent variant found in the cohort c.[1226A>G] [other].

cDNA NM_000157.4	Protein NP_000148	Protein (-39 aa)	N Alleles	%	Functional Severity
c.[1226A>G]	p.Asn409Ser	N370S	195		
Deletions			21	10.8	SEVERE 74 (37.9%)
Recombinations			16	8.2	
Insertions			16	8.2	
c.[108G>A]	p.TrpW36Ter	W(-4)X	4	2.1	
c.[256C>T]	p.Arg86Ter	R47X	4	2.1	
IVS4-2A>G +c.(-203)A>G			3	1.5	
c.[604C>T]	p.Arg202Ter	R163X	3	1.5	
c.[886C>T]	p.Arg296Ter	R257X	3	1.5	
c.[662C>T]	p.Pro221Leu	P182L	2	1.0	
c.[622C>T]	p.Gln208Ter	Q169X	1	0.5	
c.[1992C>T]	p.Arg398Ter	R359X	1	0.5	
c.[721G>A]	p.Gly241Arg	G202R	14	7.2	MODERATE 82 (42.1%)
c.[475C>T]	p.Arg159Trp	R120W	12	6.2	
c.[700G>T]	p.Gly234Trp	G195W	8	4.1	
c.[1054T>C]	p.Tyr352His	Y313H	7	3.6	
c.[1246G>A]	p.Gly416Ser	G377S	7	3.6	
c.[680A>G]	p.Asn227Ser	N188S	7	3.6	
c.[1289C>T]	p.Pro430Leu	P391L	6	3.1	
c.[517A>C]	p.Thr173Pro	T134P	4	2.1	
c.[701G>A]	p.Gly234Glu	G195E	3	1.5	
c.[887G>A]	p.Arg296Gln	R257Q	3	1.5	
c.[1124T>C]	p.Leu375Pro	L336P	3	1.5	
c.[1090G>T]	p.Gly364Trp	G325W	2	1.0	
c.[1051T>A]	p.Trp351Arg	W312R	1	0.5	
c.[1300C>T]	p.Arg434Cys	R395C	1	0.5	
c.[1304A>C]	p.Asn435Thr	N396T	1	0.5	
c.[1309G>A]	p.Val437Ile	V398I	1	0.5	
c.[1583T>C]	p.Ile528Thr	I489T	1	0.5	
c.[1604G>A]	p.Arg535His	R496H	1	0.5	
c.[1505G>A]	p.Arg502His	R463H	5	2.6	MILD 39 (20.0%)
c.[155C>T]	p.Ser52Leu	S13L	3	1.5	
c.[160G>A]	p.Val54Met	V15M	2	1.0	
c.[455G>A]	p.Gly152Glu	G113E	2	1.0	
c.[485T>C]	p.Met162Thr	M123T	2	1.0	
c.[681T>A]	p.Asn227Lys	N188K	2	1.0	
c.[706C>T]	p.Leu236Phe	L197F	2	1.0	
c.[731A>G]	p.Tyr244Cys	Y205C	2	1.0	
c.[754T>A]	p.Phe252Ile	F213I	2	1.0	
c.[1193G>A]	p.Arg398Gln	R359Q	2	1.0	
c.[437C>T]	p.Ser146Leu	S107L	1	0.5	
c.[485T>A]	p.Met162Lys	M123K	1	0.5	
c.[496G>T]	p.Asp166Tyr	D127Y	1	0.5	
c.[508C>T]	p.Arg170Cys	R131C	1	0.5	
c.[625C>T]	p.Arg209Cys	R170C	1	0.5	
c.[695G>A]	p.Gly232Glu	G193E	1	0.5	
c.[700G>T]	p.Gly234Trp	G195T	1	0.5	
c.[709A>G]	p.Lys237Glu	K198E	1	0.5	
c.[746C>T]	p.Ala249Val	A210V	1	0.5	
c.[914C>T]	p.Pro305Leu	P266L	1	0.5	
c.[928A>C]	p.Ser310Arg	S271R	1	0.5	
c.[970C>T]	p.Arg324Cys	R285C	1	0.5	
c.[1207A>C]	p.Ser403Arg	S364R	1	0.5	
c.[1208G>A]	p.Ser403Asn	S364N	1	0.5	
c.[1348T>A]	p.Phe450Ile	F411I	1	0.5	

Sequence variant nomenclature system proposed in 2000 by the Human Genome Variation Society (HGVS), different from c.[1226A>G]; [1448T>C].

**Table 3:** Coefficients of logistic regression to develop bone mineral density loss.

Variable	Coef	Std_Err	z	*p*
Intercept	−0.634942	0.216538	−2.932239	0.003365
Age at Dx	0.009794	0.006185	1.58356	0.113294
S-MRI	0.058678	0.022938	2.558053	0.010526
Splenectomy	0.678604	0.314432	2.158188	0.030913
Family History of PD	2.214037	0.388695	5.696077	1.23 × 10^−8^
Gender	0.636954	0.227262	2.802729	0.005067
Genotype *	0.455415	0.230146	1.97881	0.047837

* Genotype other than homozygous c.1226A>G vs. heterozygous c.[1226A>G]; [1448T>C].

**Table 4:** Coefficients of logistic regression to develop Parkinson’s disease.

Variable	Coef	Std_Err	z	*p*
Intercept	−2.492958	0.328728	−7.58364	3.35 × 10^−14^
Age at Dx	0.048291	0.008237	5.862482	4.56 × 10^−9^
S-MRI	−0.046485	0.029223	−1.590691	1.12 × 10^−1^
Splenectomy	1.293751	0.376888	3.432717	5.98 × 10^−4^
Family History of PD	2.214037	0.388695	5.696077	1.23 × 10^−8^
Gender	−1.052176	0.29415	−3.577003	3.48 × 10^−4^
Genotype *	2.146594	0.334419	6.418882	1.37 × 10^−10^

* Genotype other than homozygous c.[1226A>G] or heterozygous c.[1226A>G]; [1448T>C].

## Data Availability

The original contributions presented in this study are included in the article/supplementary material. Further inquiries can be directed to the corresponding author(s).

## Supplementary Materials

The following supporting information can be downloaded at: https://www.mdpi.com/article/10.3390/ijms262010088/s1.
