# Supplementary material for: Clinical Variability and Genotype–Phenotype Correlation in Spanish Patients with Type 1 Gaucher Disease: A Focus on Non-c.[1226A>G]; [1448T>C] Genotypes
Source: Int J Mol Sci. 2025 Oct 16;26(20):10088. doi: 10.3390/ijms262010088 (PMC12563535; doi:10.3390/ijms262010088)
Supplement: Supplementary file 1 [file ijms-26-10088-s001.zip › ijms-3827418-supplementary.pdf]

Supplementary material for the manuscript *"Clinical Variability and Genotype–Phenotype Correlation in Spanish Patients with type 1 Gaucher Disease: A Focus on Non-c.[1226A>G]; [1448T>C] Genotypes"*

1. *machine-learning methods used, discussing limitations and potential other analyses.*
2. Preprocessing and model specification.
   Continuous predictors were kept in their original scale; no winsorization was applied. Categorical variables were dummy-coded. We restricted model complexity to match sample size and outcome prevalence (events-per-variable principle), using penalized logistic regression (L2) when appropriate. For tree-based models we constrained maximum depth, minimum samples per split/leaf, and number of estimators to limit variance. Class imbalance (notably for PD) was handled with class-weighting in the loss function; any resampling was performed within the training folds only to avoid leakage.
3. Internal validation and hyperparameter tuning.
   To mitigate overfitting and quantify optimism, we used nested, stratified cross-validation: an inner loop for hyperparameter tuning (grid search over tree depth, min samples, and number of trees / regularization strength) and an outer loop for performance estimation. This yields nearly unbiased estimates of generalization performance for the available data. No data from the outer-test folds were used during tuning.
4. Performance metrics and calibration.
   We report AUC-ROC as the primary discrimination metric, along with accuracy, sensitivity, specificity, and F1-score with 95% CIs from bootstrap resampling. Calibration was assessed by calibration curves and Brier score; when needed, we applied Platt scaling within the cross-validation loop. Decision thresholds were selected based on Youden's J or clinically balanced sensitivity/specificity, and are reported for transparency.
5. Model interpretability.
   To enhance interpretability, we provide permutation-based variable importance for Random Forest, partial dependence profiles for the top predictors (S-MRI, age at diagnosis, genotype severity, sex), and (iii) simplified decision-tree schematics that summarize the dominant splits of the Random-Forest ensemble. These visuals are intended to explain patterns rather than to represent the full model.
6. Scope and limitations.
   All ML results reflect internal validation only on a single-center cohort (n=195). We explicitly acknowledge that external generalization is limited and that estimates may still be optimistic despite cross-validation. Consequently, ML findings are interpreted as associative and hypothesis-generating; logistic regression remains the primary inferential analysis. Code and parameter settings used for training/validation are available upon reasonable request to facilitate reproducibility.
7. Software.
   Analyses were performed in Python (scikit-learn) and R, following TRIPOD-ML guidance for transparent reporting.

Supplementary Figure S1

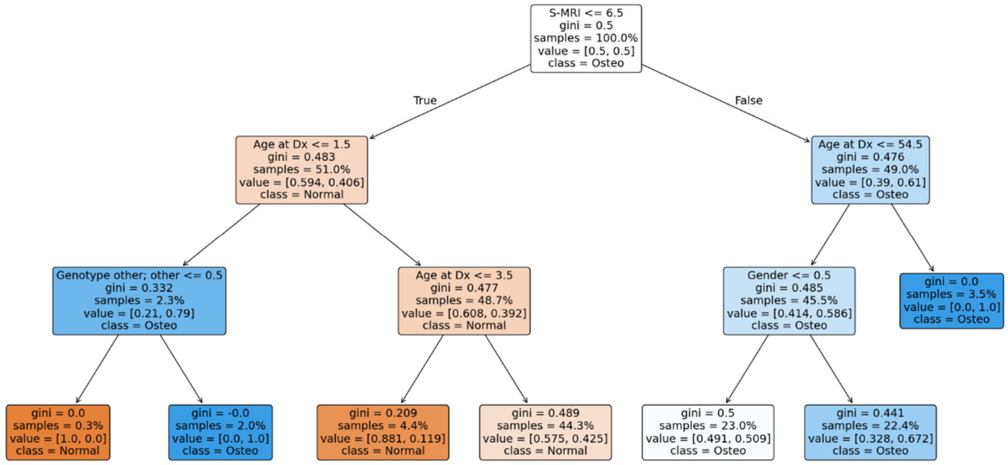

Supplementary Figure S1. Random Forest–based decision tree for prediction of bone mineral density loss in Gaucher disease patients. The main determinants identified were S-MRI score, age at diagnosis, and gender. Node color intensity represents class probability (orange = normal bone density; blue = osteopenia/osteoporosis). Gini index: impurity measure used for node splitting.

Supplementary Figure S2

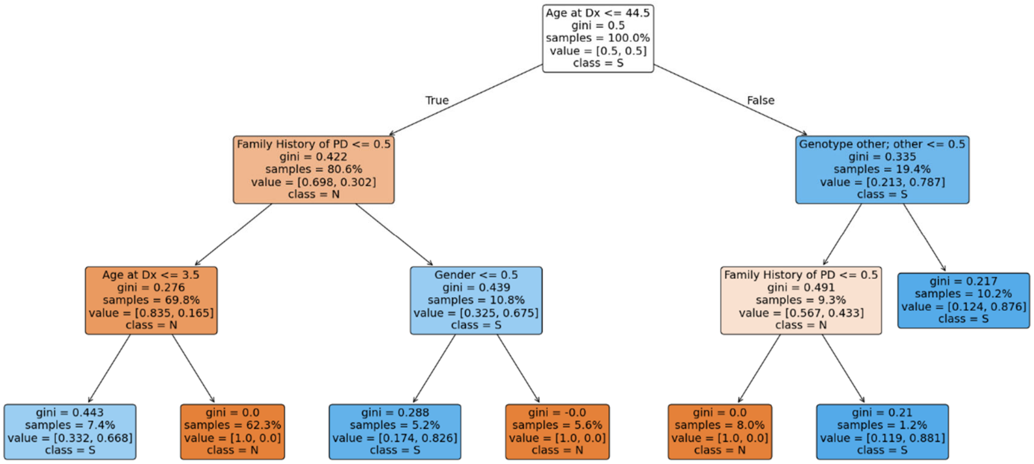

Supplementary Figure S2. Random Forest–based decision tree for prediction of Parkinson disease according to genotype, age at diagnosis, and family history. The model highlights the contribution of severe genotypes and early age at diagnosis to PD risk. Node color intensity represents class probability (orange = no PD; blue = PD). Gini index: impurity measure used to evaluate node homogeneity.
